# Diets supplemented with hydroxychloride or a hydroxychloride–organic blend of Cu, Mn, and Zn improve egg production and reduce mineral excretion in post-peak laying hens

**DOI:** 10.1016/j.psj.2026.107296

**Published:** 2026-06-14

**Authors:** Shemil P. Macelline, Sonia Y. Liu, Lane Pineda, Gavin Boerboom, Ana Isabel Garcia Ruiz, Mehdi Toghyani

**Affiliations:** a School of Life and Environmental Sciences, Faculty of Science, The University of Sydney, Sydney, New South Wales 2006, Australia; b Poultry Research Foundation, The University of Sydney, Camden, New South Wales 2570, Australia; c Trouw Nutrition, Amersfoort, Stationsstraat 77 3800 AG Amersfoort, the Netherlands

**Keywords:** Liver haemorrhage, Hydroxychloride, Laying hens, Mineral excretion

## Abstract

This study evaluated the effects of hydroxychloride (HC) and a hydroxychloride–organic (HCO) blend of trace minerals (TM), compared with inorganic sources (INO), on egg production, egg quality, mineral excretion, tissue mineral composition, and fatty liver haemorrhage (FLH) scores in laying hens from 60 to 100 weeks of age (WOA). Four dietary treatments differing in TM source were used: T1, INO TM (100 ppm Mn, 80 ppm Zn, and 12 ppm Cu supplied as inorganic salts); T2, Mn and Zn were reduced by 20 ppm and supplied as HCO; T3, the same dosage as T2 but supplied as HC; and T4, HCO supplied at half the dosage of T2 (HCO—H). A total of 320 hens were allocated to the four treatments, with 8 replicates of 10 birds each. Egg production and feed intake were recorded during 60 to 100 WOA. Egg quality parameters were measured at 60, 68, 80, and 100 WOA. Mineral composition of the tibia, eggshell, liver, and excreta was determined at 100 WOA. Trace mineral source affected egg production rate over 60–100 WOA, with hens fed HCO or HC showing higher production than those fed INO (*P* = 0.006). Hens fed HC had higher egg mass than those fed INO or HCO—H (*P* = 0.015). Eggshell breaking strength, shell thickness, Haugh unit, and tibia breaking strength were not affected by TM source (*P* > 0.05). A TM source × age interaction was observed for shell percentage (*P* = 0.002), with higher values in HCO and HC than INO at 100 WOA. As main effects, HC and HCO—H increased eggshell Cu concentration compared with INO (*P* = 0.041). Excreta Cu and Zn concentrations were lower in hens fed HCO and HC than in those fed INO, with HCO—H showing the lowest values (*P* < 0.05). FLH scores were not affected by the TM source or dose (*P* > 0.05). Overall, replacing inorganic TM with HC or HCO improved egg production, and reduced mineral excretion in post-peak laying hen.

## Introduction

Trace minerals (**TM**) are routinely supplemented in laying hens diets because feed ingredients seldom supply sufficient available zinc (**Zn**), manganese (**Mn**), and copper (**Cu**) to fully support productive performance and normal physiological function. In laying hens, these minerals are particularly important because they contribute to epithelial integrity in intestine tissues, antioxidant defence, connective tissue development, skeletal integrity, and eggshell formation. Zinc is associated with carbonic anhydrase activity and intestine epithelial maintenance, Mn is involved in proteoglycan synthesis and eggshell matrix development, and Cu contributes to collagen cross-linking and membrane integrity. Consequently, the adequacy and bioavailability of these minerals can affect laying performance, shell quality, and the general resilience of hens, especially during the post-peak period when shell quality typically declines with age (Elnesr et al., 2024; Richards et al., 2010).

Maintaining egg quality in older hens remains a major challenge for the egg industry. As hens age, eggshell thickness and shell breaking strength progressively decline, increasing the proportion of cracked and downgraded eggs and reducing the number of saleable eggs. (Duman et al., 2016; Sirri et al., 2018). This decline is multifactorial, but impaired calcium metabolism, reduced uterine function, and changes in shell ultrastructure are considered important contributing factors. Recent evidence indicates that hydroxychloride (**HC**) trace minerals may support eggshell quality in aged hens, partly through effects on uterine histological structure and inflammatory status. This suggests that mineral source may influence shell matrix formation and eggshell integrity beyond simply preventing trace mineral deficienc (Jiang et al., 2021).

In commercial layer diets, Zn, Mn, and Cu are still commonly supplied as inorganic salts. However, inorganic sources are more prone to antagonistic interactions with dietary constituents such as phytate and fibre, reducing their solubility and availability for absorption (Forbes and Erdman, 1983; Weaver and Kannan, 2001). As a result, inorganic TM are often included with a safety margin, which increases mineral excretion and raises environmental concerns, particularly for Zn and Cu. In contrast, organic and hydroxychloride sources are generally considered less reactive in feed and more available in the gastrointestinal tract, allowing lower inclusion rates without compromising productivity in many cases (Byrne and Murphy, 2022; Crosara et al., 2021; Świątkiewicz et al., 2014). A recent meta-analysis further showed that organic trace mineral supplementation can improve laying performance and egg quality traits while also reducing the environmental footprint per unit of egg output (Byrne et al., 2023).

Hydroxychloride TM have attracted increasing interest due to their crystalline structure, lower hygroscopicity, and lower reactivity compared with sulphate salts, which may improve mineral stability and delivery to sites of absorption (Sadr et al., 2025). Recent studies in laying hens have reported beneficial effects of hydroxychloride or organic TM on egg production, eggshell quality, mineral-related responses, and reproductive tissue function, particularly during late production after 60 weeks of age (Alfonso-Carrillo et al., 2024; Dong et al., 2022; Jiang et al., 2021; Palanisamy et al., 2023). However, most published work has evaluated HC and organic TM separately, rather than as a combined HC + organic (**HCO**) strategy. Therefore, it remains unclear whether the HCO blend provides additional benefits compared with HC alone. This is important because HC and organic TM may differ in mineral stability, absorption, tissue retention, and biological availability, suggesting that their combination may provide complementary effects. Another key gap is whether a reduced-dose HCO strategy remains safe and effective in very old laying hens, particularly when birds are more vulnerable to declining eggshell quality, mineral mobilisation pressure, and oxidative stress. Recent work comparing HC and organic TM suggests that different complexed sources may support similar productive outcomes, although responses can vary depending on age, trait, and dietary context (Akbari Moghaddam Kakhki et al., 2024). In broiler breeders, supplementation with an HC and organic TM blend improved egg production, egg mass, egg weight, eggshell thickness, eggshell breaking strength, and yolk colour compared with organic TM alone from 42 to 63 weeks of age (Bakhshalinejad et al., 2024). However, the potential benefits of HCO over HC alone, and the safety of half-dose HCO, have not been investigated in commercial laying hens, especially in older flocks.

Therefore, the present study evaluated inorganic TM against hydroxychloride TM and a hydroxychloride–organic blend of Zn, Mn, and Cu, offered at either conventional or reduced inclusion rates, in laying hens from 60 to 100 weeks of age. Laying performance, egg quality, mineral deposition and excretion, tibia characteristics, and fatty liver haemorrhage (**FLH**) scores were assessed. It was hypothesised that replacing inorganic TM with more bioavailable complexed sources would maintain or improve laying performance and selected egg quality traits in older hens while reducing mineral excretion, and that the hydroxychloride–organic blend may provide comparable responses even at a reduced inclusion level.

## Materials and methods

### Animal ethics statement

All the experimental procedures applied in this study were reviewed and approved by the University of Sydney Animal Ethics Committee under the ethics application number 2022/2110.

### Experimental birds and study design

A total of 320 Hy-Line Brown laying hens (56 weeks of age; **WOA**) were procured from a commercial farm (Young, NSW 2594, Australia). All hens were offered a common diet from 56 to 58 weeks to allow adaptation to the new environment. At 58 WOA, birds were randomly allocated to 32 replicate units, with each replicate consisting of 10 hens housed individually in cages measuring 25 × 50 × 50 cm and equipped with an individual feeder, nipple drinker, and pecking string. Allocation was based on body weight to ensure comparable average body weight across all replicates while minimising cumulative variance between replicate units. The 32 replicate units were then randomly assigned to four dietary treatments in a completely randomized design, with eight replicates per treatment. The period from 58 to 60 WOA was considered an adaptation period to the experimental diets, during which egg production was monitored to confirm that all birds were actively laying. The lighting program consisted of 16 h of light and 8 h dark every 24 h. Egg production and feed intake data were recorded from 60 to 80, and 80 to 100 WOA, and then totaled for the entire 40 weeks period.

Four dietary treatments differing in trace mineral (**TM**) source were used (Table 1). Treatment 1 was an inorganic trace mineral (**INO TM**) diet containing 100 ppm Mn, 80 ppm Zn, and 12 ppm Cu supplied as sulphate. In Treatment 2, Mn and Zn were reduced by 20 ppm and supplied as hydroxychloride-organic blend (**HCO**). Treatment 3 had the same inclusion levels as Treatment 2, but the minerals were supplied as hydroxychloride (**HC**). Treatment 4 contained HCO supplied at half the dosage of Treatment 2 (**HCO—H**). The HC and HCO mineral premixes were supplied by Trouw Nutrition. The HC premix was supplied as Selko® IntelliBond® hydroxychloride trace minerals, while the HCO premix was supplied as IntelliOpt®, a trace mineral blend based on IntelliBond® hydroxychloride trace minerals and Optimin® organic trace mineral (Trouw Nutrition, Amersfoort, the Netherlands).

**Table 1:** Mineral composition specifications in each premix used in experimental diets.

Mineral source	Composition of mineral premix, g/kg					
	Mn	Zn	Cu	Fe	I	Se
Inorganic (sulphate)	100	80	12	15.0	1.0	0.30
Hydroxychloride + Organic blend	80	60	12	15.0	1.0	0.30
Hydroxychloride	80	60	12	15.0	1.0	0.30
Hydroxyxhloride + Organic blend (Half dose)	40	30	6.0	7.5	0.5	0.15

In all four treatments, Fe, I and Se were supplemented as inorganic form (FeSO_4_, KI and Na_2_SeO_3_).

### Experimental diet preparation

Diets were based on wheat, barley, soybean meal, rice bran, and canola meal (Table 2), and their nutrient specifications were evaluated by near-infrared spectroscopy using the AMINOIR Advanced programme (Evonik operations GmbH, Hanau, Germany) prior to feed formulation. Each diet was mixed separately and offered in mash form. The birds were fed in two feeding phases, such as 60 to 80 weeks and 80 to 100 weeks.

**Table 2:** Ingredient composition and nutrient specifications of basal diets for phases 1 and 2.

Ingredient (g/kg)	Phase 1 (60 to 80 weeks)	Phase 2 (80 to 100 weeks)
Wheat	550.70	555.70
Barley	100.00	100.00
Soybean meal	85.00	70.70
Rice bran	80.00	100.00
Canola meal	50.00	50.00
Limestone grit	79.20	90.20
Limestone fine	20.0	10.0
Mono calcium phosphate	4.50	2.00
Canola oil	17.00	8.33
Sodium bicarbonate	3.50	3.35
Vitamin premix^1^	1.00	1.00
Mineral premix^2^	1.00	1.00
Salt	1.25	1.00
Choline chloride	0.50	0.50
Lysine HCl	2.30	2.39
*dl*-Methionine	1.85	1.67
*l*-Threonine	0.85	0.77
*l*-Isoleucine	0.71	0.58
*l*-Valine	0.41	0.22
Xylanase^3^	0.10	0.10
Phytase^4^	0.03	0.03
*Nutrient specifications (g/kg, unless otherwise specified)*		
Dry matter	914.1	916.7
AME, kcal/kg	2,740	2,710
Crude protein	142.9	141.0
Crude fat	44.2	39.4
Starch	411	415
Crude Fibre	32.7	34.1
Calcium	40.5	41.0
Total phosphorus	5.60	5.10
Available phosphorus	3.50	3.30
Sodium	1.90	1.80
Chloride	1.80	1.70
Potassium	6.00	5.80
Lysine^5^	6.85	6.62
Methionine	3.86	3.64
Methionine +Cysteine	6.23	6.02
Threonine	4.79	4.59
Isoleucine	5.48	5.22
Tryptophan	1.53	1.50
Arginine	7.46	7.32
Valine	6.03	5.73

1 Vitamin Premix: retinol 12 MIU, cholecalciferol 5 MIU, tocopherol 50 mg/kg, menadione 3 mg/kg, thiamine 3 mg/kg, riboflavin 9 mg/kg, pyridoxine 5 mg/kg, cobalamin 0.025 mg/kg, niacin 50 mg/kg, pantothenate 18 mg/kg, folate 2 mg/kg, biotin 0.2 mg/kg.

2 Compositions of mineral premixes in each experimental diet are presented in Table 1,.

3 Xylanase was added as RONOZYME® WX 2000 (CT) at 100 g/T, providing 200 FXU/kg of complete feed.

4 Phytase was added as Axtra® PHY 10 G at 30 g/T, providing 300 FTU/kg of complete feed.

5 Amino acids are presented in digestible basis.

### Data collection and chemical analysis

Birds were individually weighed at 60, 80, and 100 weeks of age, while feed intakes were calculated for 60 to 80 weeks, 80 to 100 weeks, and 60 to 100 weeks of age. Mortalities were recorded as they occurred and totalled for the entire period. The cause of death was recorded in consultation with the university veterinarian. Egg production was recorded daily to calculate the egg production rate. Hen-day egg production was calculated using the number of hen-days for each feeding phase and the entire experiment period, and the number of eggs laid during the same period. Egg weights were recorded weekly from the eggs laid in 48 hrs and then averaged for the week. Egg production data and egg weights were used to calculate egg mass in each replicate (Egg mass = Egg weight, g × egg production, %). Feed conversion ratio (FCR) was defined as the feed intake required to produce 1 g of egg mass. FCR=Feedintake/Eggmass

External and internal egg quality parameters were assessed at weeks 60, 68, 80, and 100 of the feeding trial, using 25% of eggs collected from each replicate. Parameters measured included eggshell breaking strength, eggshell weight, albumen weight, yolk weight (expressed as a percentage of egg weight), yolk colour score, and Haugh unit (HU). Eggs were first weighed, after which eggshell breaking strength (N) was determined at the broad end using a 3-point bending test with a texture analyzer (Perten TVT 6700, Stockholm, Sweden) fitted with a 75-mm cylindrical probe. Eggs were then carefully broken onto a flat glass surface positioned on a metal stand above a reflective mirror for internal quality assessment. The height of the thick albumen was measured with an albumen height gauge (Technical Services and Supplies, York, United Kingdom). The HU was calculated as: HU = 100 × log [(h + 7.57) − (1.7 × w0.37)] where h (mm) = albumen height and w (g) = egg weight (Monira et al., 2003). Albumen and yolk were carefully separated using a plastic scraper, weighed, and expressed as percentages of whole egg weight. Yolk color was scored using a DSM Yolk Color Fan (DSM, Switzerland, 2005). Eggshell membranes were removed, and the shells were washed, air-dried, weighed, and expressed as a percentage of whole egg weight. At the conclusion of the trial (week 100), two birds per replicate (16 birds per treatment) were randomly selected, euthanized, and decapitated. The right tibia and liver were collected, weighed, and used for mineral and bone strength analysis. Tibia breaking strength (N) was determined at the mid-shaft as the peak fracture force using a texture analyzer (Perten TVT 6700) fitted with a breaking probe. All bones were positioned in the same orientation, and force was applied at mid-length. The broken tibiae, liver samples, and eggshells (from weeks 60, 80, and 100) were analyzed for ash content. Samples were dried at 105°C for 24 h, ignited at 600°C for 8 h, cooled in a desiccator, and weighed. Pooled faecal samples from each replicates were collected at week 100 to analyze mineral excretion. Mineral compositions of diets, eggshells, tibiae, liver, and faeces were analyzed at the University of New South Wales using inductively coupled plasma optical emission spectrometry (ICP-OES; PerkinElmer OPTIMA 7300, PerkinElmer Inc., Waltham, MA), following digestion with nitric acid and hydrogen peroxide as described by Hopcroft et al. (2020).

At 100 WOA, the same two euthanized birds per replicate were used for the following assessments, which were conducted blind to treatment allocation. The skin over the breast region was removed to expose the breast muscle and keel bone for visual assessment. Keel bone curvature was evaluated using a 4-point scoring system, as described by Muir et al. (2022). A score of 1 indicated a straight keel, score 2 indicated mild curvature, score 3 indicated moderate curvature, and score 4 indicated severe keel curvature (Hy-Line International, 2016). Breast muscle condition was also assessed using a 4-point scale (Hy-Line International, 2019), where score 0 represented a very lean or cachectic breast muscle, score 1 indicated a slightly concave breast contour, score 2 represented an ideal breast muscle condition, and score 3 indicated abundant or slightly excessive breast muscle.

The liver was subsequently assessed for FLHS according to the 6-point scoring system described by Shini et al. (2019). A score of 0 represented a liver with normal appearance and no haemorrhage, score 1 indicated 1 to 10 subcapsular petechial or ecchymotic haemorrhages, and score 2 indicated more than 10 subcapsular petechial or ecchymotic haemorrhages. Scores of 3 or higher were assigned to livers showing prominent haematomas, extensive haemorrhage, and rupture of the liver capsule.

### Statistical Analysis

Performance, tissue mineral content, and tibia breaking strength data were subjected to one-way ANOVA analysis as a completely randomized design, using the General Linear Model procedure of JMP Pro 16.0 software package. Each replicate (8 cages of one bird each) per treatment was considered as an experimental unit, and the values presented in the tables are the means with the pooled standard error of mean (SEM). When a significant effect of treatment was detected, Tukey’s HSD test was used to make pairwise comparisons between treatment means. Significance was set at *P* < 0.05. Egg quality parameters were subjected to analysis as a 4 × 4 factorial arrangement using a 2-way ANOVA of GLM procedure to assess the main effects (mineral inclusion and laying age) and any 2-way interactions. Liver, breast and keel scores were analysed using the Wilcoxon non-parametric test to obtain mean scores. The significnat effect of treatment were determined at Probability>Chisqure. When significnat, scores means were separated using Steel-Dwass All Pairs test.

## Results

According to the data presented in Table 3, the analyzed mineral contents of the premixes and diets generally achieved the intended trace mineral profiles. Table 4 summarises the effect of dietary treatments on egg production parameters over different periods. Dietary treatments did not influence egg weight, feed intake and FCR for any production periods (*P* > 0.05). Egg production rate was influenced by dietary treatments for 60 – 80 weeks (*P* = 0.007) and 60 – 100 weeks (*P* = 0.006) but was not affected for the 80 – 100 weeks period (*P* = 0.121). During the 60 – 80 week period, the egg production rates in INO, HCO, and HC were not statistically different, whereas HCO—H exhibited a lower egg production rate than HCO and HC by approximately 3.90%. The egg production rates of HCO and HC were higher than INO and HCO—H for a 60 – 100 week period. Egg mass was influenced by dietary treatments for 60 – 80 weeks and 60 – 100 weeks (*P* = 0.015) but not for the 80 – 100 weeks period (*P* = 0.214). HCO and HC exhibited higher egg mass compared to INO and HCO—H in both the 60 – 80 and 60 – 100 week periods.

**Table 3:** Analyzed mineral composition in premixtures and experimental diets.

Premixes	Ca g/kg	Cu g/kg	Fe g/kg	K g/kg	Mn g/kg	Na g/kg	P g/kg	Zn g/kg
Inorganic	7.43	13.9	13.4	5.51	96.1	1.12	2.77	79.3
Hydroxychloride + Organic	1.51	14.5	20.3	5.85	75.5	0.94	2.19	58.7
Hydroxychloride	2.22	11.3	14.3	2.43	77.6	0.62	0.82	59.5


Diets (phase 1)	Ca %	Cu ppm	Fe ppm	K %	Mn ppm	Na %	P %	Zn ppm
INO	3.97	18.1	184	0.67	179	0.18	0.56	135
HCO	3.75	17.3	196	0.65	161	0.19	0.57	121
HC	4.02	20.9	185	0.68	159	0.16	0.58	118
HCO—H	4.11	12.7	164	0.64	122	0.18	0.58	82

Diets (phase 2)								

INO	4.15	16.6	168	0.63	164	0.14	0.53	128
HCO	4.24	18.3	160	0.64	146	0.15	0.55	112
HC	4.22	18.8	174	0.62	152	0.13	0.55	117
HCO—H	4.14	13.3	153	0.61	123	0.14	0.53	77

INO: Inorganic; HCO: Hydroxychloride + Organic; HC: Hydroxychloride; HCO—H: HCO half a dose.

**Table 4:** Effects of dietary treatments on egg production parameters, feed intake and feed conversion ratio from 60 to 80 weeks, 80 to 100 weeks and 80 to 100 weeks.

Treatment	Egg weight g/egg			Egg production rate %			Egg mass g egg/bird			Feed intake g/bird/d			FCR g/g		
	60-80	80-100	60-100	60-80	80-100	60-100	60-80	80-100	60-100	60-80	80-100	60-100	60-80	80-100	60-100
INO	66.1	67.8	66.8	92.8^ab^	79.4	86.5^b^	61.3^bc^	53.8	57.8^b^	133.6	122.4	128.4	2.183	2.276	2.223
HCO	66.2	67.3	66.6	94.9^a^	81.6	88.6^a^	62.8^ab^	54.8	59.1^ab^	132.3	121.5	127.2	2.107	2.220	2.156
HC	66.7	67.8	67.2	95.0^a^	82.2	89.0^a^	63.4^a^	55.7	59.8^a^	134.4	122.8	129.1	2.121	2.205	2.158
HCO—H	66.6	67.7	67.0	91.3^b^	79.8	85.9^b^	60.8^c^	54.1	57.6^b^	130.5	124.0	127.4	2.148	2.294	2.212
SEM	0.469	0.442	0.378	0.809	0.941	0.684	0.610	0.702	0.521	1.021	2.256	1.331	0.027	0.046	0.030
*P*-value	0.734	0.766	0.748	0.007	0.121	0.006	0.015	0.214	0.015	0.057	0.888	0.749	0.237	0.469	0.283

a-bMeans in a column not sharing a superscript are significantly different (Tukey test at *P* < 0.05).

INO: Inorganic; HCO: Hydroxychloride + Organic; HC: Hydroxychloride; HCO—H: HCO half a dose.

The interactive effect of dietary treatments and birds' age on egg quality parameters is presented in Table 5. There were dietary treatment and age interactive effects observed on yolk colour (*P* = 0.002) and eggshell relative weight (*P* = 0.002). Birds' age and dietary treatments interaction on egg yolk colour showed that dietary treatments did not influence the yolk colour at 60, 68, and 80 weeks of age, but at week 100, HC had a lower yolk colour index than INO and HCO—H but comparable to HCO. The interaction effect on the relative weights of eggshell showed that dietary treatments did not influence the eggshell weights at 60, 68, and 80 weeks of age, whereas INO exhibited lighter eggshell % compared to HCO and HC diets at 100 weeks of age. As main effects, dietary treatments did not influence shell breaking strengths (*P* = 0.471), eggshell thickness (*P* = 0.120), Haugh unit (*P* = 0.870), albumen (*P* = 0.494) and yolk weights (*P* = 0.263). However, birds’ age as a main effect influenced eggshell breaking strength (*P* = 0.003), eggshell thickness (*P* < 0.001), Haugh unit (*P* = 0.005), and relative weight of albumen (*P* = 0.001). The transition of the bird’s age from 60 to 100 weeks reduced eggshell breaking strength by 8.3% (38.6 versus 42.1 N), eggshell thickness by 19.4% (0.383 versus 0.475 mm), Haugh unit by 9.64% (78.7 versus 87.1), and increased the relative weight of albumen by 1.6% (64.3% versus 63.3%).

**Table 5:** Effects of dietary treatments on egg quality parameters at different ages.

Item		Eggshell		Yolk Colour	Haugh Unit	Egg component (%)		
Diet	Age (wk)	BS^1^	Thickness			Shell	White	Yolk
INO	60	44.6	0.479	11.4^ab^	89.1	10.0^abc^	63.7	26.3
	68	45.7	0.472	10.6^ab^	88.8	10.6^a^	62.4	27.1
	80	39.4	0.382	12.4^a^	79.8	10.0^abc^	62.2	27.9
	100	37.6	0.375	9.7^bc^	76.0	8.6^e^	65.0	26.4
HCO	60	40.2	0.491	11.3^ab^	88.5	10.4^a^	63.9	25.7
	68	41.7	0.476	9.8^bc^	84.1	10.0^abc^	63.8	26.2
	80	41.9	0.416	11.9^a^	85.4	10.0^abc^	63.4	26.6
	100	38.2	0.385	8.3^cd^	77.9	9.4^bcd^	63.4	27.2
HC	60	40.2	0.468	11.4^ab^	86.9	10.0^abc^	63.4	26.6
	68	44.3	0.475	9.9^bc^	87.7	10.4^bc^	62.7	27.0
	80	45.5	0.390	10.7^ab^	82.3	10.2^bc^	63.4	26.5
	100	40.4	0.391	7.9^d^	82.5	9.4^bcd^	64.7	25.9
HCO—H	60	43.3	0.460	9.6^bc^	83.8	10.5^a^	62.1	27.4
	68	43.7	0.465	10.7^ab^	86.7	10.2^ab^	63.1	26.7
	80	40.2	0.392	11.8^a^	82.9	10.0^abc^	62.8	27.2
	100	38.3	0.379	9.9^bc^	78.3	8.9^de^	64.1	27.0
SEM		1.87	0.011	0.392	3.36	0.178	0.577	0.549
Main effects								
Diet								
INO		41.8	0.427	11.0	83.4	9.78	63.3	26.9
HCO		40.5	0.442	10.3	84.0	9.92	63.6	26.4
HC		42.6	0.431	10.0	84.9	10.00	63.5	26.5
HCO—H		41.4	0.424	10.5	82.9	9.89	63.0	27.1
Age (weeks)								
60		42.1^a^	0.475^a^	10.9	87.1^a^	10.23	63.3^b^	26.5
68		43.8^a^	0.472^a^	10.3	86.8^a^	10.27	63.0^b^	26.7
80		41.8^a^	0.395^b^	11.7	82.6^ab^	10.03	63.0^b^	27.0
100		38.6^b^	0.383^b^	8.9	78.7^b^	9.06	64.3^a^	26.6
*P*-value								
Diet		0.471	0.120	0.003	0.870	0.426	0.494	0.263
Age		0.003	<.001	<.001	0.005	<.001	0.001	0.555
Diet × age		0.298	0.774	0.002	0.794	0.002	0.100	0.328

Means are based on 25% of eggs tested at each time point.

1 Shell breaking strength (N), shell thickness (mm) a-bMeans in a column not sharing a superscript are significantly different (Tukey test at *P* < 0.05) INO: Inorganic; HCO: Hydroxychloride + Organic; HC: Hydroxychloride; HCO—H: HCO half a dose.

The interactive effect of dietary treatments and birds’ age on mineral composition of eggshells is presented in Table 6. No diet and age interaction effect was found on the composition of the tested minerals in eggshells (*P* > 0.05). As the main effect, diet only influenced Cu content in eggshells (*P* = 0.041), where HC and HCO—H supported higher Cu concentration than INO by 43.6% and 35.5%, respectively. As a main effect, age influenced Ca, Fe, K, Mg, Mn, P, and Zn concentrations in eggshells (*P* < 0.05). Transitioning from 60 to 100 weeks of age, the concentrations of Ca increased by 3.5% (410 versus 396 g/kg), Fe by 42% (3.18 versus 2.20 mg/kg), Mg by 17.4% (4.19 versus 3.57 g/kg), and P by 4.1% (1.28 versus 1.23 g/kg). In contrast, transitioning from 60 to 100 weeks of age, the eggshell concentrations of K decreased by 17.7% (0.93 versus 1.13 g/kg), Mn by 52% (0.12 versus 0.25 mg/kg), and Zn by 24.3% (0.84 versus 1.11 mg/kg) .

**Table 6:** Effects of dietary treatments and age of the birds on eggshell mineral composition.

Item		Ca, g/kg	Cu, mg/kg	Fe, mg/kg	K, g/kg	Mg, g/kg	Mn, mg/kg	Na, g/kg	P, g/kg	Zn, mg/kg
Diet	Age (wk)									
INO	60	395	0.61	2.26	1.17	3.65	0.25	1.73	1.25	1.1
	80	404	0.57	0.92	0.99	3.63	0.17	1.56	1.14	0.65
	100	412	0.68	2.61	0.98	4.2	0.12	1.64	1.34	0.77
HCO	60	395	0.77	2.18	1.18	3.55	0.25	1.73	1.24	1.1
	80	402	0.91	0.97	0.98	3.62	0.21	1.56	1.2	0.74
	100	408	0.74	2.28	0.93	4.15	0.11	1.53	1.27	0.78
HC	60	396	0.9	2.21	1.08	3.59	0.26	1.63	1.22	1.13
	80	401	1.04	0.84	1.05	3.74	0.2	1.66	1.2	0.77
	100	407	0.74	3.97	0.92	4.29	0.11	1.61	1.32	0.91
HCO—H	60	397	0.78	2.17	1.09	3.51	0.25	1.66	1.21	1.11
	80	399	0.91	0.91	1.1	3.62	0.21	1.73	1.18	0.63
	100	411	0.84	3.61	0.88	4.12	0.14	1.53	1.21	0.91
SEM		2.7	0.121	0.578	0.069	0.094	0.027	0.087	0.032	0.112
Main effects										
Diet										
INO		404	0.62^b^	1.93	1.04	3.83	0.18	1.65	1.25	0.84
HCO		402	0.81^ab^	1.81	1.03	3.77	0.19	1.61	1.24	0.87
HC		401	0.89^a^	2.34	1.02	3.88	0.19	1.64	1.24	0.94
HCO—H		402	0.84^a^	2.23	1.02	3.75	0.20	1.64	1.20	0.88
Age (weeks)										
60		396^c^	0.76	2.20^b^	1.13^a^	3.57^b^	0.25^a^	1.69	1.23^b^	1.11^a^
80		401^b^	0.86	0.91^a^	1.03^b^	3.65^b^	0.20^b^	1.63	1.18^c^	0.69^b^
100		410^a^	0.74	3.12^c^	0.93^c^	4.19^a^	0.12^c^	1.58	1.28^a^	0.84^b^
*P*-value										
Age		<0.001	0.397	<0.001	<0.001	<0.001	<0.001	0.951	<0.001	<0.001
Diet		0.769	0.041	0.643	0.969	0.363	0.771	0.196	0.329	0.756
Age × Diet		0.745	0.768	0.652	0.639	0.981	0.957	0.579	0.196	0.962

a-bMeans in a column not sharing a superscript are significantly different (Tukey test at *P* < 0.05).

INO: Inorganic; HCO: Hydroxychloride + Organic; HC: Hydroxychloride; HCO—H: HCO half a dose.

Table 7 presents the effects of dietary treatments on the mineral composition of excreta at 100 weeks of age. Dietary treatments significantly influenced excreta concentrations of Cu (*P* = 0.028) and Zn (*P* = 0.047). Compared with INO, Cu concentrations in excreta were reduced by 20.8% and 25.6% in layers offered HCO and HC diets, respectively, while HCO—H resulted in a greater reduction of 56.2%. For Zn, excreta concentrations did not differ significantly among INO, HCO, and HC; however, HCO—H decreased Zn concentration by 36.8% compared with INO.

**Table 7:** Effects of dietary treatments on mineral composition in excreta determined at 100 weeks of age.

Diet	Ca g/kg	Cu mg/kg	Fe g/kg	K g/kg	Mg g/kg	Mn g/kg	Na g/kg	P g/kg	Zn g/kg
INO	257	317^a^	3.77	90.1	43.3	2.56	12.11	6.71	1.06^a^
HCO	262	251^b^	4.13	73.0	34.3	2.11	12.80	5.30	0.93^ab^
HC	267	236^b^	3.60	87.0	41.7	2.25	9.71	5.98	0.91^ab^
HCO—H	268	139^c^	2.85	83.8	36.7	1.72	8.55	5.72	0.67^b^
SEM	15.8	39.4	0.705	9.23	41.1	0.251	1.544	7.33	0.093
*P*-value	0.957	0.028	0.630	0.590	0.386	0.141	0.195	0.588	0.047

a-b Means in a column not sharing a superscript are significantly different (Tukey test at *P* < 0.05).

INO: Inorganic; HCO: Hydroxychloride + Organic; HC: Hydroxychloride; HCO—H: HCO half a dose.

Table 8, Table 9 present the effects of dietary treatments on liver and tibia mineral composition, respectively, in laying hens at 100 weeks of age. No significant differences were observed among the dietary treatments for mineral composition in either the liver or tibia. Table 10 presents the effects of dietary treatments on tibia breaking strength, fat pad weight, liver weight, breast muscle weight, keel curvature scores, and eggshell colour scores measured at 100 weeks of age. Dietary treatments did not significantly affect any of these parameters (*P* > 0.05)

**Table 8:** Effects of dietary treatments on mineral composition in liver determined on 100 weeks of age.

Diet	Ca g/kg	Cu mg/kg	Fe g/kg	K g/kg	Mg g/kg	Mn mg/kg	Na g/kg	P g/kg	Zn g/kg
INO	2.26	16.0	0.99	47.5	2.71	40.6	13.8	44.2	0.42
HCO	1.42	16.3	1.61	47.1	2.61	36.8	13.9	43.3	0.40
HC	1.60	13.8	0.82	39.6	2.24	32.4	11.4	36.1	0.30
HCO—H	1.47	14.3	1.10	53.1	2.86	36.9	13.7	46.8	0.39
SEM	0.439	2.61	0.166	6.33	0.364	4.65	1.93	6.10	0.063
*P*-value	0.507	0.880	0.513	0.526	0.664	0.668	0.765	0.641	0.559

INO: Inorganic; HCO: Hydroxychloride + Organic; HC: Hydroxychloride; HCO—H: HCO half a dose.

**Table 9:** Effects of dietary treatments on mineral composition in tibia ash determined on 100 weeks of age.

Diet	Ca g/kg	Cu mg/kg	Fe g/kg	K g/kg	Mg g/kg	Mn mg/kg	Na g/kg	P g/kg	Zn g/kg
INO	371	0.99	0.16	4.65	5.89	26.7	10.8	162.4	0.38
HCO	368	0.92	0.15	4.28	5.78	26.9	10.5	161.6	0.40
HC	368	0.97	0.15	4.65	5.81	26.2	10.8	162.2	0.40
HCO—H	367	0.85	0.16	4.45	5.89	25.0	10.6	161.3	0.39
SEM	2.9	0.112	0.007	0.160	0.104	2.02	0.12	1.32	0.015
*P*-value	0.835	0.828	0.667	0.300	0.809	0.914	0.197	0.940	0.868

INO: Inorganic; HCO: Hydroxychloride + Organic; HC: Hydroxychloride; HCO—H: HCO half a dose.

**Table 10:** Effects of dietary treatments on tibia breaking strength, fat pad weight, liver, breast muscle and keel curvature scores determined at 100 weeks of age.

	Parametric analysis			Non-parametric analysis		
Diet	Tibia BS^1^	Fat pad (g/kg)	Eggshell colour	Liver score^2^	Breast score	Keel score
INO	203	61.3	75.3	17.3	17.1	12.3
HCO	211	61.4	76.1	13.3	15.1	17.5
HC	200	64.4	75.3	14.3	14.8	18.5
HCO—H	208	55.8	75.4	21.2	19.0	17.7
SEM	10.20	0.449	1.08			
P-value	0.871	0.604	0.942	0.247	0.460	0.470

1 Breaking strength (N).

2 fatty liver haemorrhages scores a-b Means in a column not sharing a superscript are significantly different (Tukey test at *P* < 0.05) INO: Inorganic; HCO: Hydroxychloride + Organic; HC: Hydroxychloride; HCO—H: HCO half a dose.

## Discussion

Published research evaluating combined organic and HC TM supplementation in commercial laying hens remains limited. In broiler breeders, an HC–organic blend of Zn, Cu, and Mn was reported to support egg production and egg quality, at levels broadly comparable to organic TM supplementation alone (Bakhshalinejad et al., 2024). In contrast, several studies in laying hens have evaluated HC or organic TM separately and reported beneficial or comparable effects on egg production eggshell quality, and reproductive tissue function compared with INO sources (Akbari Moghaddam et al., 2024; Dong et al., 2022; Olukosi et al., 2019; Qiu et al., 2020). However, whether an HCO blend produces responses comparable to, or different from, HC alone in old commercial laying hens remains unclear. A recent broiler study reported positive responses to blends of HC and organic Zn on body weight and feed conversion ratio (Sadr et al., 2024). In this context, the present study is novel in evaluating the effects of a combined HC and organic source of Mn, Zn, and Cu in older laying hens, relative to conventional inorganic trace minerals. The present study showed that both HCO and HC improved egg production over the 60 to 100 week period, while only HC improved egg mass compared to the inorganic TM. In addition, the complexed mineral sources reduced excreta Cu concentration, while the half-dose HCO treatment also lowered excreta Zn. However, mineral source did not influence eggshell breaking strength, shell thickness, Haugh unit, liver mineral composition, tibia mineral composition, or tibia breaking strength. Therefore, under the conditions of the present study, the main advantage of HC and HCO was improved long-term productive performance and mineral excretion rather than a broad improvement in all egg quality traits.

In the present study, egg weight was not influenced by dietary TM strategy. This was expected, as egg weight is primarily regulated by dietary crude protein, amino acid supply, and energy intake, rather than trace mineral source or concentration when diets are otherwise nutritionally adequate (Réhault-Godbert et al., 2019). In addition, the concentrations of Zn, Cu, and Mn in whole eggs are relatively low, reported as 5.15, 0.24, and 0.17, respectively (Heflin et al., 2018), which may further explain the limited response of egg weight to TM supplementation observed in the present study. The higher egg production rate observed in hens fed HCO and HC, and the higher egg mass observed in hens fed HC, are consistent with previous studies reporting that alternative TM sources can support productive performance in late-phase hens (Dong et al., 2022; Jiang et al., 2021; Palanisamy et al., 2023). These findings are also broadly aligned with the meta-analysis of Byrne et al. (2023), which reported that organic TM may support layer performance and sustainability outcomes compared with inorganic TM sources. A possible explanation is that alternative TM sources may support mineral-related processes involved in eggshell formation, including eggshell ultrastructure, shell gland function, and enzyme activity, particularly in older hens, where tissue integrity and physiological resilience become increasingly challenged (Dong et al., 2022; Qiu et al., 2020).

The responses to HCO were not consistent across all productive traits. Although HCO increased egg production rate, the clearest egg mass response was observed with HC. This suggests that responses to different TM strategies may depend on mineral source, inclusion level, physiological endpoint, and duration of feeding. The HCO—H treatment also provided useful insight into the response to reduced TM inclusion. Despite receiving lower supplemental Zn, Mn, and Cu, hens fed HCO—H maintained egg production and egg mass at levels broadly comparable to those fed the INO treatment. This suggests that more available TM sources can partially compensate for lower inclusion rates, which is consistent with previous reports that organic or hydroxychloride sources can replace inorganic TM at reduced dietary levels without compromising productivity (Crosara et al., 2021; Świątkiewicz et al., 2014).

A clear practical outcome of the alternative TM strategies in the present study was the reduction in excreta Cu concentration with HCO and HC, with the greatest reduction observed in HCO—H. These differences were unlikely to be explained by variation in feed intake, as dietary treatments did not significantly affect feed intake. Although excreta mineral concentration alone does not allow direct estimation of mineral retention or balance, the lower excreta Cu concentration is environmentally relevant because excessive Cu and Zn output is a recognised limitation of high inorganic mineral feeding programs. Similar reductions in mineral excretion with complexed TM sources have been reported previously in poultry and are considered an important practical argument for their commercial use (Byrne et al., 2023; Dozier et al., 2003; Świątkiewicz et al., 2014). The more pronounced excretion response for Cu than for Zn or Mn may reflect mineral specific differences in digestive interactions, intestinal transport, and post-absorptive regulation. For example, phytate can strongly chelate divalent trace minerals, including Cu, Zn, and Mn, which may influence their solubility and availability within the gastrointestinal tract (Reddy et al., 1982).

Despite the improved long-term productive performance observed with HCO and HC, neither source improved shell breaking strength or shell thickness. Instead, both traits declined with advancing age, which is consistent with the established literature on older laying hens (Bain et al., 2016; Fu et al., 2024). Age-related deterioration in eggshell quality is driven largely by reduced efficiency of calcium transport, shell gland function, and eggshell formation capacity, rather than trace mineral supply alone. Jiang et al. (2021) reported that HC trace minerals improved some shell related responses in aged hens, partly through effects on uterine structure and cytokine expression; however, such responses may not be sufficient to overcome the broader calcium related and physiological constraints associated with very old hens. Therefore, although more available TM sources may support shell matrix formation and tissue integrity, maintaining shell strength in older hens likely requires a broader nutritional strategy that considers calcium supply, calcium solubility, limestone particle size, and vitamin D status, in addition to TM source.

The diet × age interaction for shell percentage was noteworthy. At 100 WOA, hens fed HCO and HC had higher shell percentage than those fed INO, although shell thickness and shell breaking strength were not affected. This suggests that the complexed TM sources may have supported shell deposition or shell membrane development sufficiently to increase the relative shell contribution to total egg weight, but not to a level that translated into measurable improvements in shell mechanical strength. Because shell strength depends not only on shell quantity, but also on shell ultrastructure, membrane integrity, crystal organisation, and calcium deposition efficiency (Fu et al., 2024; Ren et al., 2023), the higher shell percentage observed at 100 WOA should be interpreted as a modest structural response rather than evidence of full protection against age related shell deterioration.

The diet × age interaction for yolk colour also deserves further consideration. Yolk pigmentation depends primarily on the intake, absorption, transport, and deposition of dietary carotenoids, particularly xanthophylls, into the yolk (Dansou et al., 2023; Yunitasari et al., 2023). Because the basal diets were the same apart from the mineral premix, it is unlikely that the lower yolk colour observed at 100 weeks in HC, and numerically also in HCO, was due to a direct pigmenting effect of the trace minerals themselves. A more plausible explanation is that the improved egg mass in HC, and the numerically greater productive output in HCO, diluted the available yolk pigments across a greater quantity of egg output. In other words, when dietary pigment intake remains constant, hens producing more egg mass may deposit less pigment per unit of yolk, resulting in lower yolk colour scores. This explanation should be viewed as a biological inference rather than a directly measured mechanism in the present study, but it is consistent with the known dependence of yolk colour on carotenoid intake and deposition dynamics. From a practical perspective, these findings suggest that pigment inclusion may need to be adjusted when highly productive mineral programs are used in older hens, particularly when maintaining a specific commercial yolk colour target is important.

In the present study, FLH score was not affected by dietary TM source or level. This suggests that, under the conditions of this experiment, replacing INO with HCO or HC was not sufficient to alter the severity of visible hepatic haemorrhagic lesions in very old hens. This is not unexpected, as FLH is a multifactorial metabolic disorder influenced by hepatic lipid accumulation, positive energy balance, reproductive status, body weight, feed efficiency, oxidative stress, and overall liver metabolic function, rather than trace mineral supply alone (Anene et al., 2023; Shini et al., 2019; Tu et al., 2023). Therefore, although Zn and Cu are involved in antioxidant defence and lipid related metabolic pathways, the absence of a treatment effect on FLH score indicates that the TM strategies used in the present study did not measurably influence gross liver lesion severity.

The absence of dietary effects on liver and tibia mineral concentrations should be interpreted cautiously. Tissue mineral concentrations are subject to homeostatic regulation and may be less responsive than performance or excreta mineral measurements for detecting responses to dietary TM strategy over a long laying cycle (Broom et al., 2021). Previous studies have also shown that changes in fecal mineral excretion may occur without consistent changes across all tissue mineral pools, indicating that tissue deposition and excretion responses do not necessarily follow the same pattern (Qiu et al., 2020; Yang et al., 2021). Therefore, the lack of treatment effects on liver and tibia mineral composition does not necessarily indicate an absence of biological response; rather, it suggests that the most practically relevant responses in the present study were expressed through egg production, excreta Cu concentration, and shell Cu concentration, rather than through gross mineral accumulation in liver or tibia.

## Conclusion

Overall, the present findings indicate that replacing INO Zn, Mn, and Cu with HC or HCO improved long term laying performance in post-peak hens and reduced excreta Cu concentration. However, these benefits did not extend to all eggshell quality traits, as shell thickness and shell breaking strength continued to decline with advancing age. In addition, the lower yolk colour observed in the more productive HC and HCO treatments at 100 WOA may have reflected pigment dilution associated with greater egg mass output. Therefore, in older hens, complexed TM strategies should be viewed as one component of a broader nutritional approach that can support productive persistence and reduce mineral output, but are unlikely on their own to fully prevent age related declines in eggshell quality.

## Authorship contribution

**Shemil P. Macelline:** Investigation, Methodology, Data curation, Formal analysis, Writing - original draft, Writing - review & editing. **Sonia Yun Liu**: Conceptualization, Methodology, Writing - review & editing. **Lane Pineda:** Conceptualization, Methodology, Writing - review & editing. **Gavin Boerboom:** Methodology, Writing - review & editing. **Ana Isabel Garcia Ruiz:** Methodology, Writing - review & editing. **Mehdi Toghyani:** Conceptualization, Methodology, Feed formulation, Funding acquisition, Project administration, Supervision, Writing - review & editing.

## Disclosures

The authors declare that this study received financial support from Trouw Nutrition, a commercial entity whose products (hydroxychloride and organic trace minerals) were evaluated in the research. While the funding organization provided financial support for the project, they had no role in the data collection, analysis and interpretation of the results. The authors have no other financial or personal relationships with individuals or organizations that could inappropriately influence the work reported in this paper.
